# A New Minimally Invasive Technique for Thoracolumbar/Lumbar Focal Kyphosis Due to Osteoporotic Vertebral Fracture: A Case Report

**DOI:** 10.7759/cureus.66069

**Published:** 2024-08-03

**Authors:** Masato Tanaka, Abd El Kader Al Askar, Chetan Kumawat, Shashank J Ekade, Koji Uotani

**Affiliations:** 1 Department of Orthopedic Surgery, Okayama Rosai Hospital, Okayama, JPN; 2 Department of Orthopedic Surgery, Okayama University Hospital, Okayama, JPN

**Keywords:** oblique lumbar interbody fusion, novel technique, c-arm free, thoracolumbar focal kyphosis, osteoporotic vertebral fractures

## Abstract

Osteoporotic vertebral fractures are common fractures in the elderly population and are often associated with low back pain and disruption in daily living activities. Reconstruction surgeries, such as corpectomy, are among the treatment options for these conditions. However, a corpectomy requires a longer surgical procedure and involves a significant amount of blood loss. We present the case of an 80-year-old woman with severe low back pain due to an L2 fracture and focal kyphosis treated with a novel minimally invasive technique. The patient underwent anterior and posterior surgery in the right decubitus position using a C-arm-free technique. Hyperlordotic cages were inserted in the upper and lower disc space via a lateral approach, while percutaneous pedicle screws were inserted from a posterior approach. These procedures were performed simultaneously under navigation guidance only.

## Introduction

Osteoporotic vertebral fractures (OVFs) are very common fractures in the elderly population. After a severe wedged fracture, patients may experience low back pain, pulmonary dysfunction, and disturbances in activities of daily living [1]. Usually, conservative treatments for OVFs, such as pain management, physiotherapy, and orthosis, are effective in most cases.

Severe OVFs occur when the fractured vertebral body height collapses to less than one-third of its original height. These patients may experience intractable back pain, focal kyphosis deformities, progressive neurologic impairment, additional complicated morbidities, and even a heightened mortality risk. According to the literature, conservative treatment for such patients is ineffective [2].

A wide variety of techniques and approaches have been described for the treatment of these fractures. The surgical goals include correction of deformity, restoration of collapsed vertebrae, maintenance of sagittal balance, and achieving ideal bone fusion with stable internal fixation. Among these, reconstruction surgeries such as corpectomy, pedicle subtraction osteotomy (PSO), and vertebral column resection are considered for addressing focal kyphosis [3]. However, these procedures are often associated with significant challenges, including extensive surgical time, considerable blood loss, and the risk of long-term complications like proximal junctional kyphosis or structural failures [4].

Given the complexities and ongoing debates surrounding the optimal surgical intervention and the selection of fusion techniques and instrumentation [5], this discourse introduces a novel surgical technique. This new approach is tailored for patients experiencing thoracolumbar/lumbar focal kyphosis as a result of severe OVF, a promising improvement over traditional methods.

## Case presentation

This research was approved by the ethics committee of our institution (approval number 472), and informed consent from the patient undergoing surgery was duly obtained.

Patient history

An 80-year-old woman presented to our hospital with severe lower back pain and gait disturbance. She had sustained a severe OVF at L2 six years prior. Prior to her visit, the patient had undergone long-term conservative treatment for pain management, which proved ineffective. Her medical history included paroxysmal supraventricular tachycardia (diagnosed in 1999), lumbar canal stenosis (diagnosed in 2019), and osteoporosis (diagnosed in 2021). Teriparatide was used for her osteoporosis treatment.

Physical examination

During the examination, she showed no neurological deficits. However, due to severe back pain, she was unable to walk more than 200 meters. Her Visual Analogue Scale (VAS) score for low back pain was 73 mm, and her Oswestry Disability Index (ODI) was 53.3%.

Preoperative imaging

**Figure 1:**
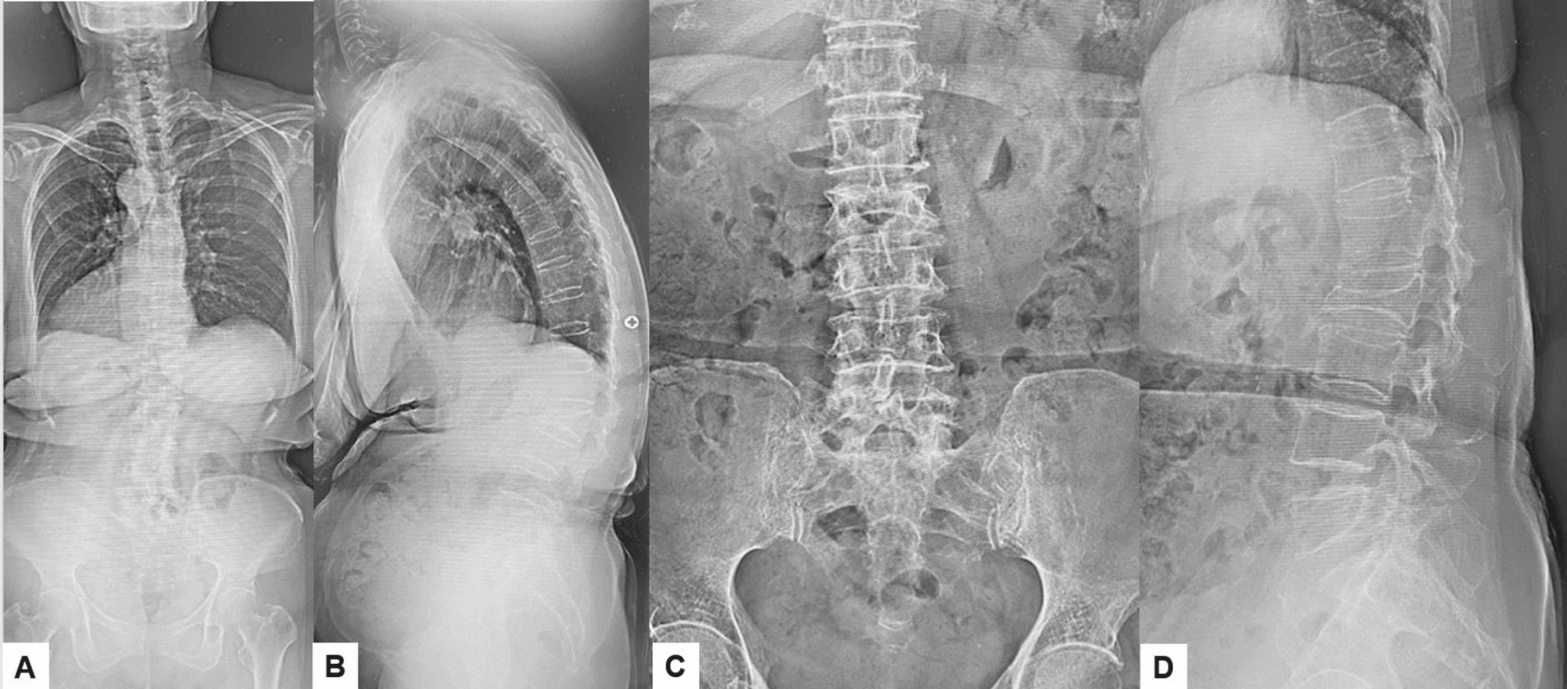
Preoperative spinal radiograms (A) Posteroanterior radiogram. (B) Lateral spinal radiogram. (C) Anteroposterior lumbar radiogram. (D) Lateral lumbar radiogram.

**Figure 2:**
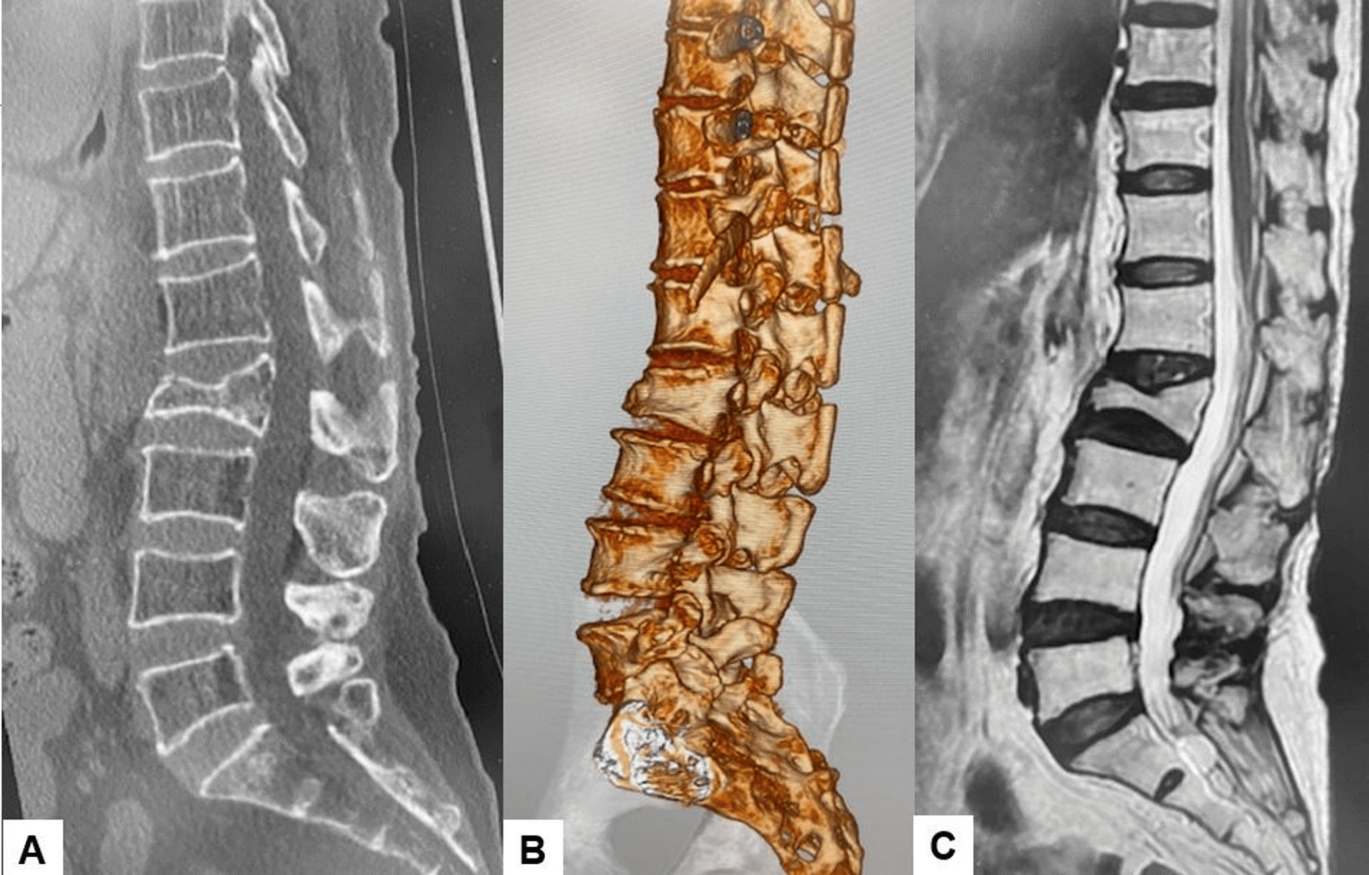
Preoperative CT and MRI (A) Mid-sagittal reconstruction CT. (B) Three-dimensional reconstruction CT. (C) T2-weighted mid-sagittal MRI.

Preoperative spinal radiographs revealed severe sagittal malalignment, with the L2 vertebra collapsed and a focal kyphosis between L1 and L3 measuring 34 degrees. The bone mineral density was 0.623 g/cm², and the T-score for the lumbar vertebrae was 3.5 SD (Figures 1, 2).

Surgery

**Figure 3:**
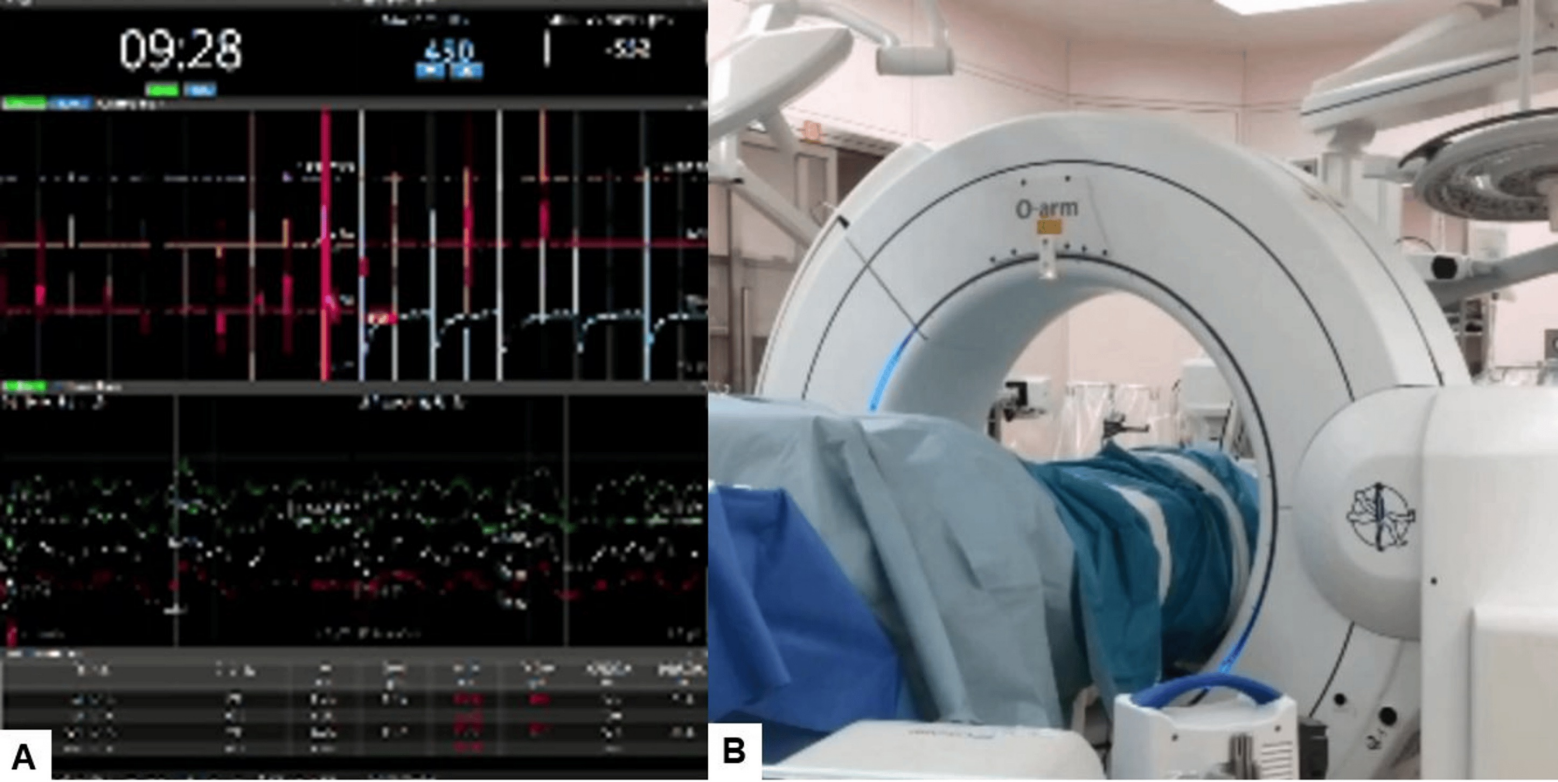
Neuromonitoring and O-arm (A) Continuous neuromonitoring was initiated at the beginning of the procedure. (B) A CT scan was obtained using the O-arm to provide detailed imaging guidance.

The procedure employed a simultaneous anterior and posterior approach, performed in a single position using the right lateral decubitus position. This method avoided the use of a C-arm, thereby minimizing radiation exposure. Continuous neuromonitoring was initiated at the start of the procedure to ensure the patient’s neurological safety throughout (Figure 3A).

Following sterilization and draping, a reference frame was carefully inserted into the left sacroiliac joint. A CT scan was then obtained using the O-arm to provide detailed imaging guidance (Figure 3B). A precise 4 cm lateral skin incision was made, guided by a navigational probe. This incision allowed for the sequential dissection of the external, internal, and transverse abdominal muscles, providing access to the surgical site.

**Figure 4:**
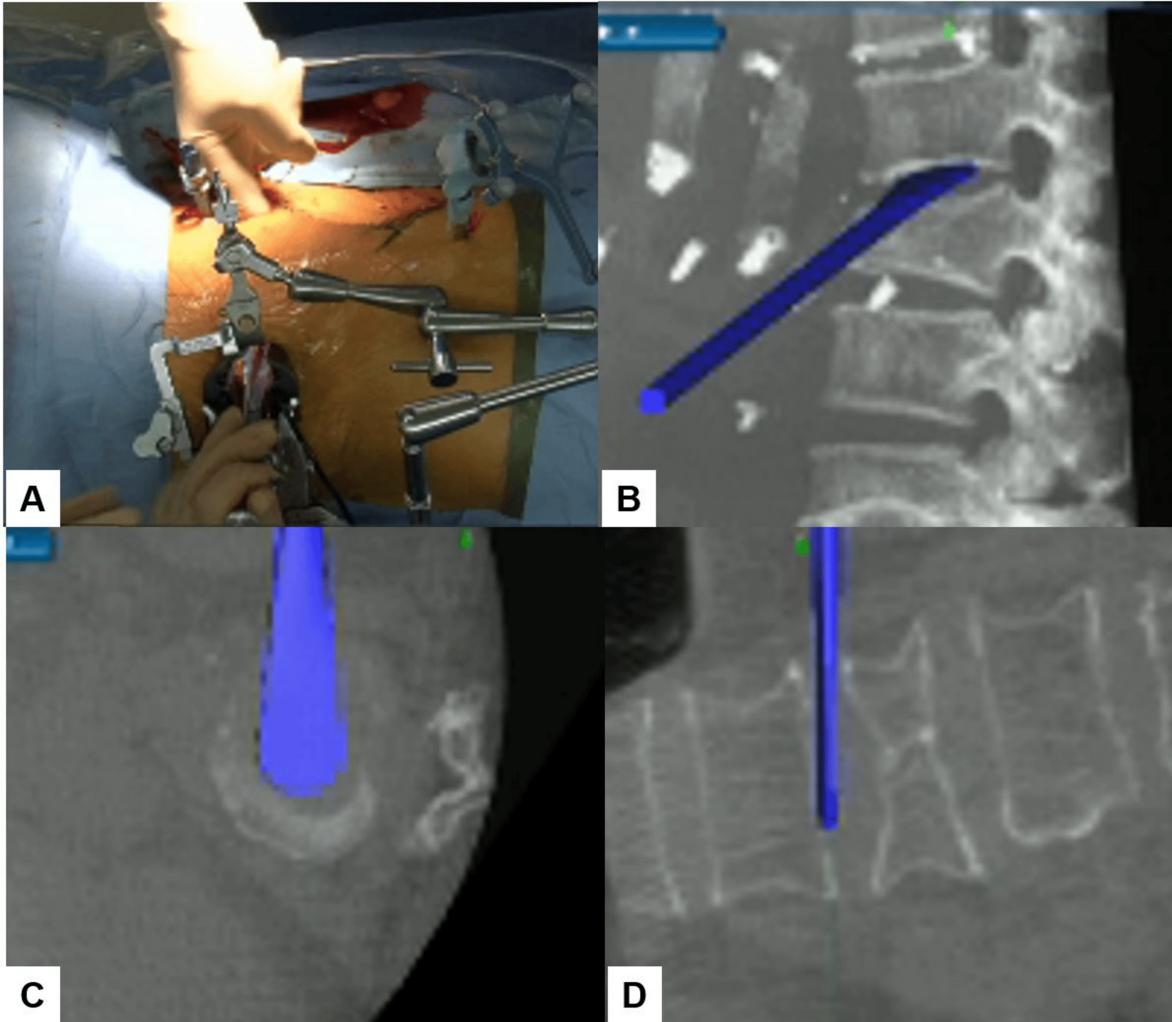
Navigated shaver and Cobb elevator (A) Intraoperative image. (B) Sagittal view. (C) Axial view. (D) Coronal view. A navigated Cobb elevator was used to remove the disc.

After registering each surgical instrument, the L1/2 and L2/3 disc spaces were accurately exposed with the aid of a navigation probe. A self-retaining retractor with illumination was then placed to maintain clear visibility and access to the operative field. Discectomy at the L1/2 level was meticulously performed using a knife and navigated shavers (Figure 4A, 4B), followed by complete disc removal with a navigated Cobb elevator (Figure 4C, 4D).

**Figure 5:**
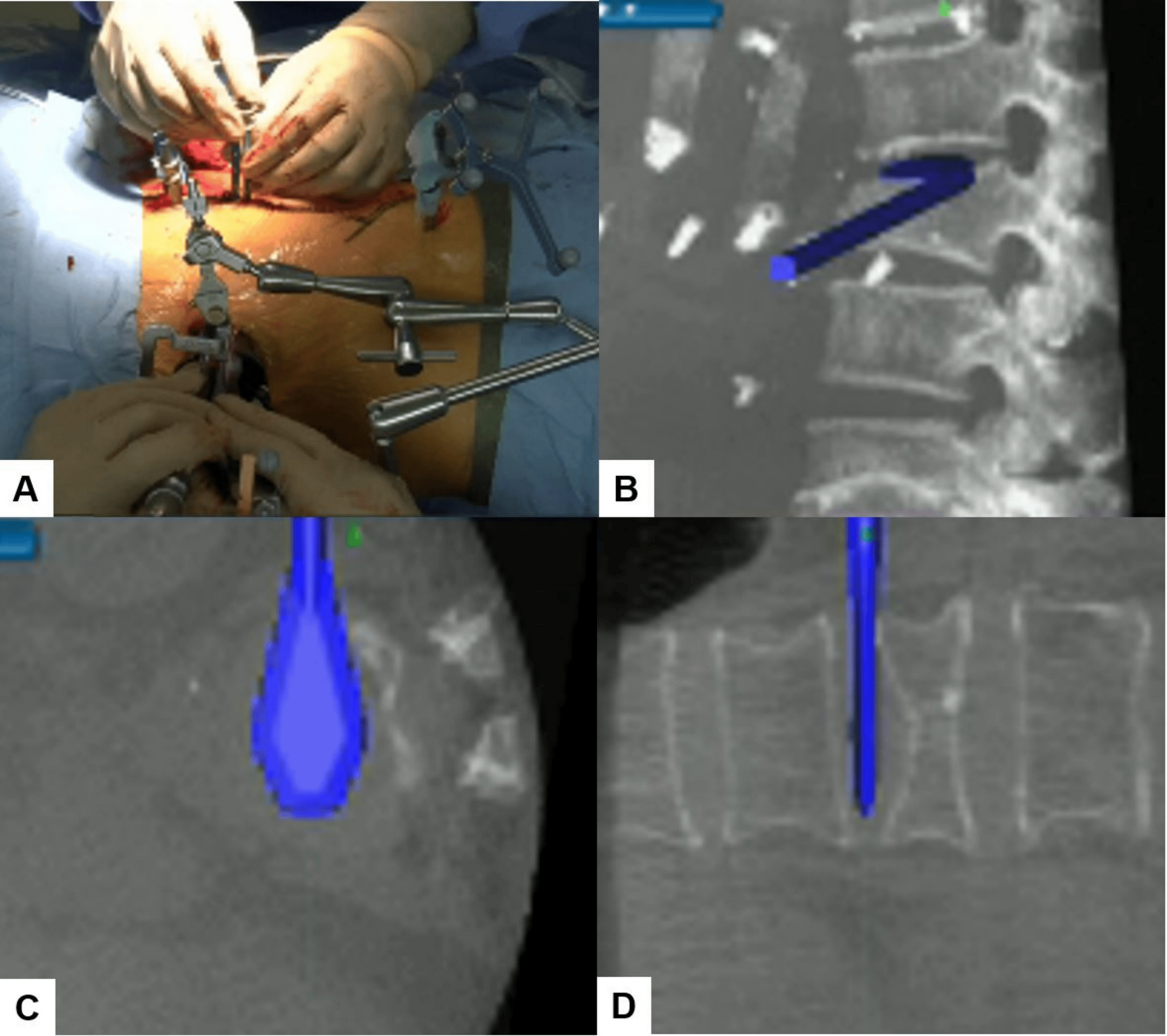
Navigated spreader (A) Intraoperative image. (B) Sagittal view. (C) Axial view. (D) Coronal view. A navigated spreader was used carefully to expand the disc space.

**Figure 6:**
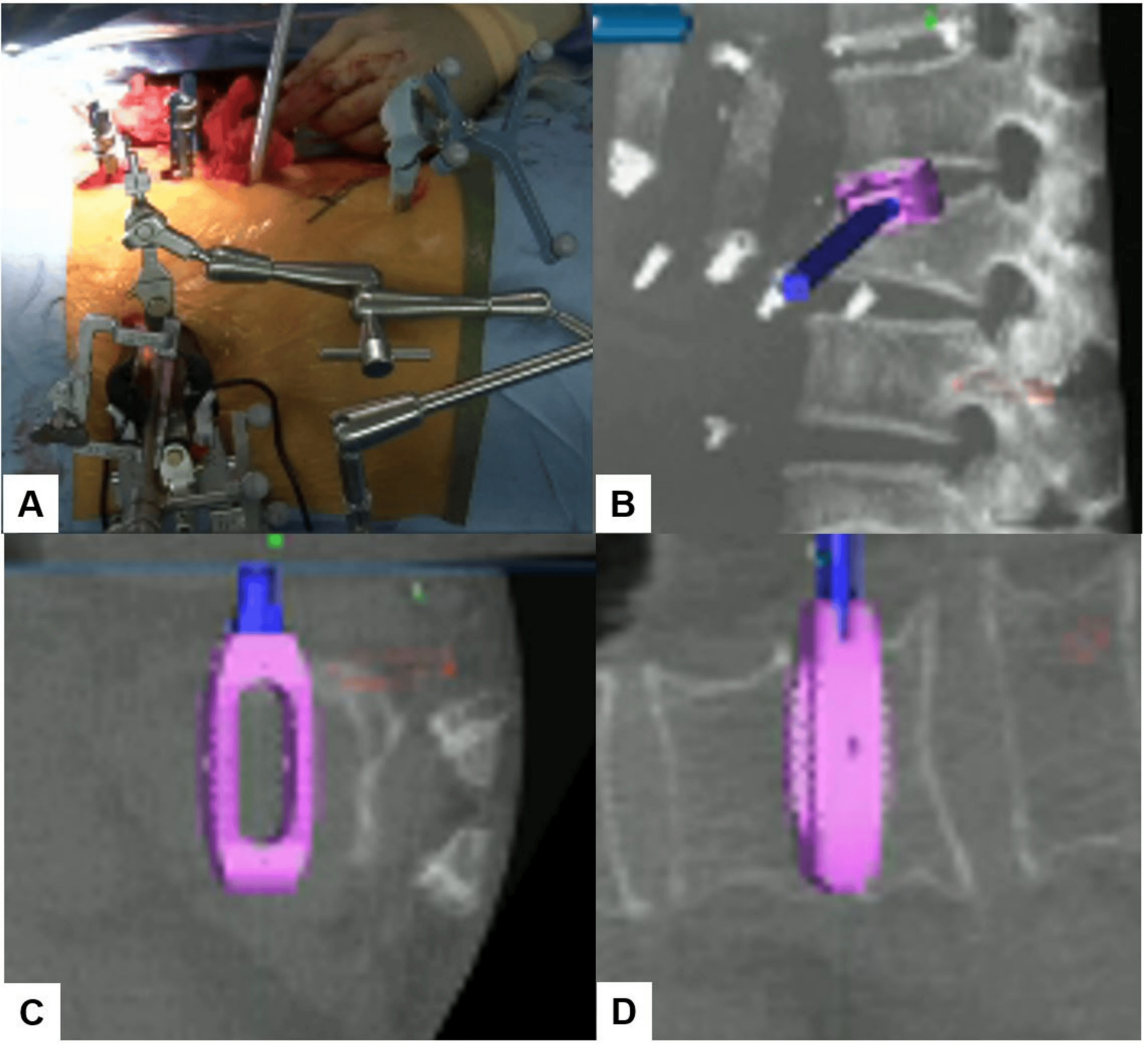
Navigated cage (A) Intraoperative image. (B) Sagittal view. (C) Axial view. (D) Coronal view. A hyperlordotic cage was inserted under the guidance of navigation.

Subsequently, the disc space was carefully expanded using a navigated spreader and trial (Figure 5). This precise preparation allowed for the insertion of a hyperlordotic cage (Clydesdale™ PTC spinal system), angled at 12 degrees, under the guidance of navigation to ensure optimal placement (Figure 6). Following this, the same meticulous steps were repeated for the insertion of the second cage.

**Figure 7:**
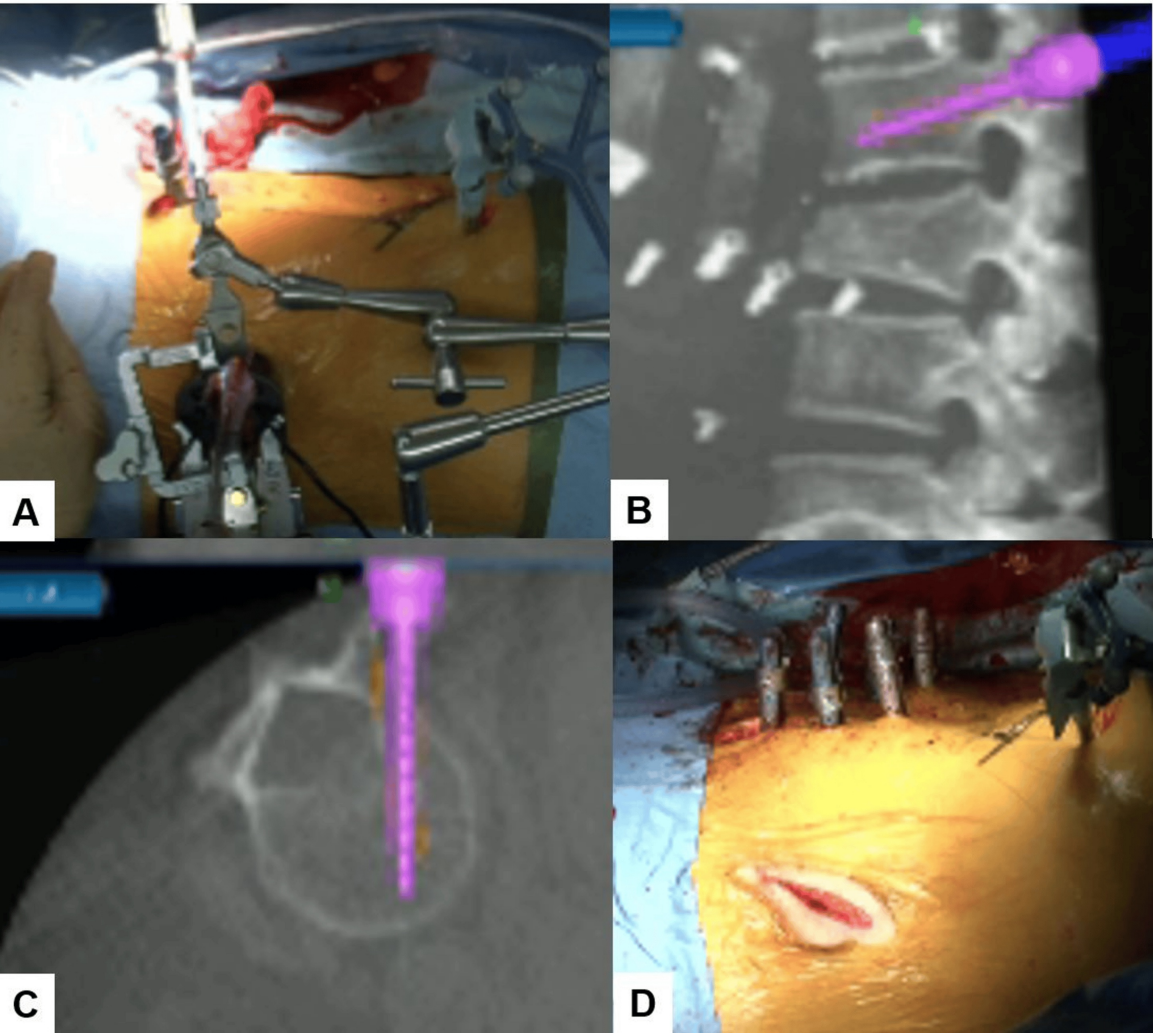
PPS insertion (A) Intraoperative image. (B) Sagittal view. (C) Axial view. (D) Intraoperative post-screw placement image. A second surgeon simultaneously inserted PPS during the insertion of the hyperlordotic cages. PPS, percutaneous pedicle screws

During the insertion of the cages, a second surgeon simultaneously inserted percutaneous pedicle screws (PPS) from a posterior approach (Figure 7). Each pedicle screw required only a 1.8 cm skin incision. A critical technique involved inserting the cranial screws first to maintain the accuracy of the navigation system. The sequence of insertion was as follows: first, the cranial PPS was inserted; next, a cranial hyperlordotic cage was placed; then, the middle and distal PPS were inserted; and finally, a distal hyperlordotic cage was positioned. Following the successful placement of six screws across L1, L2, and L3, rods were inserted percutaneously. Compression forces were then applied through the screws to enhance lordosis, ensuring optimal spinal alignment and curvature correction.

**Figure 8:**
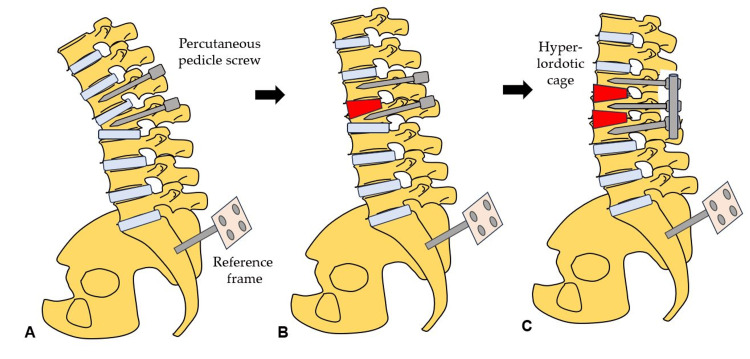
Steps of the dual cage technique (A) Insertion of cranial PPS. (B) Simultaneous insertion of a cranial hyperlordotic cage and PPS. (C) Insertion of a caudal hyperlordotic cage and rods and compression. PPS, percutaneous pedicle screws

The steps of this novel technique are illustrated in Figure 8 to provide a clearer understanding of the procedure.

Postoperative imaging

**Figure 9:**
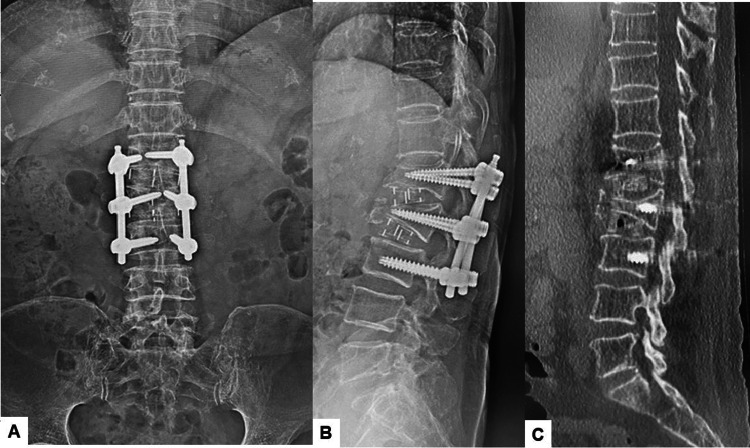
Postoperative radiograms and CT (A) Anteroposterior lumbar radiogram. (B) Lateral lumbar radiogram. (C) Mid-sagittal reconstruction CT.

Postoperative radiographs and CT scans confirmed the successful realignment of the spine. The focal kyphosis angle between L1 and L3 was corrected from 34 degrees to 14 degrees. CT showed that the hyperlordotic cages and screws were precisely inserted (Figure 9).

Clinical results

The surgery was completed successfully, with a total operative time of 133 minutes and an estimated blood loss of 100 ml. At the one-year follow-up, the patient was walking smoothly without any gait disturbance. The VAS for low back pain improved from 73 mm to 13 mm, and the ODI decreased from 53.3% to 13.3%. No major or severe complications were reported.

## Discussion

Osteoporosis is recognized as a significant global health concern. One of the most prevalent complications associated with this condition is OVFs, which can lead to chronic pain, disability, spinal malalignment such as focal kyphosis, and consequently, functional decline and diminished quality of life for elderly patients. OVFs are predominantly located in the thoracolumbar region [6]. While usually OVFs result in mild symptoms and can be treated conservatively, approximately 30-40% of these fractures present with severe symptoms, necessitating surgical intervention. Indications for surgery include instability at the fracture site, post-traumatic kyphosis, intractable pain unresponsive to conservative treatments, neurological deficits, and significant spinal canal stenosis [7].

Thoracolumbar/lumbar focal kyphosis is a common cause of sagittal spinal malalignment. The importance of sagittal plane balance in the spine cannot be overemphasized, as it is associated with good long-term functional outcomes. Compression of the fractured vertebral body and exacerbation of the kyphotic deformity result in forward movement of the body’s center of gravity and an increase in the sagittal vertical axis [6]. When evaluating kyphotic deformities, it is important to distinguish between global and focal types. Mild global kyphosis deformities may be managed conservatively or sometimes require isolated posterior column osteotomies [6]. In contrast, patients with fixed focal kyphotic deformities typically present with severe pain, early fatigue, forward-leaning, and difficulty maintaining horizontal gaze and may require more invasive methods depending on the desired correction.

In our patient, percutaneous balloon kyphoplasty and percutaneous vertebroplasty are not applicable due to the nature of the fracture. The fracture is old and consolidated, meaning that the bone has healed and solidified over time, making these procedures ineffective for addressing the condition [8]. Regarding osteotomy techniques for correcting kyphotic deformities, several methods have been reported in the literature [9], including Pontes osteotomy, Smith-Peterson osteotomy, PSO, and combined anterior-posterior correction. While these techniques can achieve the desired correction, they also pose significant risks and serious complications, such as dural tears, nerve root injuries, and spinal cord injuries, which can be disastrous [10]. Additionally, complications such as fixation failure, kyphosis recurrence, prolonged surgical time, and significant blood loss are associated with these techniques. Long-term corrective fusion, such as from the thoracic spine to pelvis fixation, is an alternative option. However, this option has limitations for elderly patients because of its high mortality rate (2.4%) and high complication rate (up to 70%) [11]. It is important to consider that the majority of these patients are elderly and have multiple comorbidities, so minimizing risks is paramount.

Combined anterior corpectomy and posterior fixation procedures have been observed to be associated with higher complication rates, regardless of whether the procedures are performed in one or two stages. These complications include severe blood loss, prolonged surgical time, nerve root and spinal cord injuries, and wound infections [12]. Additionally, in corpectomy procedures, non-union rates and cage subsidence are high [13]. Furthermore, cage placement can be challenging, especially when using wide-footprint cages, which may result in cumbersome insertion processes [14]. One study demonstrated that the mean surgical time for minimally invasive corpectomy and posterior fixation was 275 minutes, which was significantly less than conventional open surgery [8]. However, in our study, the surgical time was less than half of that reported by that study, totaling only 133 minutes. One of the most important advantages of our technique is that there is no need for a C-arm. The implantation of the expandable cage via lateral approach is another good option for this focal kyphosis. In the near future, this technique will be performed under navigation guidance.

It has been reported that correcting kyphotic deformities completely with a posterior-only approach, without anterior support, can be challenging. Dobran et al. also noted that using combined surgery in patients with focal kyphosis could facilitate fusion development and alignment more effectively [15]. However, in our case, we chose not to pursue these techniques due to our patient’s advanced age and multiple comorbidities. Instead, we aimed to provide her with the best possible treatment while minimizing potential risks. The expandable cage is a good alternative for this condition. However, the expandable cage from the posterior approach and the facet joints have to be removed. A lateral approach can be used for a lateral expandable cage, but that is not navigated.

This is the first study to reveal details about focal kyphosis correction and improvement in sagittal balance following the minimally invasive procedure using double hyperlordotic cages with a C-arm-free technique. In our patient, surgery was performed in the lateral decubitus position to avoid the need for repositioning and redraping between anterior and posterior procedures. This approach helped to reduce operative time, the risks of contamination, and the inconvenience of re-registration for navigation purposes. Hiyama et al. reported an additional average repositioning time of 34 minutes between lateral decubitus and prone positions [16]. Although percutaneous posterior pedicle screw fixation in the lateral position using C-arm fluoroscopy can be technically challenging, navigation technology makes it feasible and accurate [17]. Additionally, the lateral position is generally better tolerated by patients compared to prone surgery and avoids potential concerns associated with prone positioning, such as postoperative vision loss, cardiovascular complications, hypovolemia, reduced pulmonary compliance, and cardiac arrest [18]. One of the most important risks of this technique is cage subsidence. To prevent this, teriparatide was used for this patient. However, a fracture of the upper instrumented vertebra occurred (Table 1).

**Table 1:** Various techniques for OVF and fixed focal kyphosis OVF, osteoporotic vertebral fracture; PSO, pedicle subtraction osteotomy; VCR, vertebral column resection

Technique	Deformity correction	Surgical invasiveness	Surgical time	Blood loss	Complication
Ballon kyphoplasty	No	Little	Very short	Very few	Cement leakage
Ponte osteotomy	Intermediate	Middle	Intermediate	Intermediate	Proximal junctional kyphosis
PSO	Intermediate	High	Long	High	Neurological deterioration
Posterior VCR	Complete	Very high	Very long	Very high	Neurological deterioration
Long spinal fusion (T10-pelvis)	Intermediate	Very high	Very long	Very high	Mortality rate: 2.4%; complication rate: up to 70%)
Corpectomy	Intermediate	Middle	Long	High	Cage subsidence, dislodge, non-union
Dual hyperlordotic cages	Intermediate	Middle	Intermediate	Intermediate	Cage subsidence

Extended surgical times for elderly patients with multiple serious comorbidities can pose significant hazards. At times, procedures may need to be staged, leading to prolonged hospitalization and delayed mobilization, which carries its own set of risks. In our technique, the surgical time was 133 minutes and the blood loss was 100 ml, significantly less than the mean reported for other procedures. For example, Suk et al. compared anterior-posterior surgery versus closing wedge osteotomy for kyphotic OVFs and reported mean blood loss of 2,892 mL for the former and 1,930 mL for the latter [19]. Postoperatively, our patient experienced notable improvements, with the VAS for low back pain decreasing from 73 mm to 13 mm and the ODI improving from 53.3% to 13.3%.

Placing the cage in the correct position typically necessitates the use of fluoroscopy. However, in our technique, the double hyperlordotic cages are navigated, allowing for three-dimensional visualization in all planes on the navigation monitor. This approach significantly reduces radiation exposure to the surgeon and operating room staff in terms of fluoroscopy time and exposure [20].

There are several limitations to our new technique. Further studies with a larger population and a longer follow-up duration are needed to accurately evaluate the outcomes of this technique. Secondly, there is a risk of intraoperative CT-based surgical errors, particularly those involving the misplacement of spinal implants due to inadvertent movement of the reference frame. Lastly, due to the simultaneous nature of the technique, it requires the involvement of two surgeons to perform the procedure.

## Conclusions

The simultaneous use of double hyperlordotic cages with PPS is a useful technique that reduces surgical time and intraoperative blood loss. Hyperlordotic cages were inserted in the upper and lower disc space via a lateral approach, while PPS were inserted from a posterior approach. Additionally, acceptable corrections can be achieved with this minimally invasive technique. Navigation was useful to achieve precise cages and screw insertions without fluoroscopy. This novel navigation technique yields promising outcomes for patients with thoracolumbar focal kyphosis due to severe OVFs.
